# Enhanced Yield of *GmJAG1*-Edited Soybeans Accompanied by Improved Function of the Rhizosphere Microbiome

**DOI:** 10.3390/plants15121828

**Published:** 2026-06-12

**Authors:** Xiuping Chen, Chenhui Hou, Huilin Yu, Jiajian Xie

**Affiliations:** State Key Laboratory for Biology of Plant Diseases and Insect Pests, Institute of Plant Protection, Chinese Academy of Agricultural Sciences, Beijing 100193, China; xpchen@ippcaas.cn (X.C.); hou13213938029@163.com (C.H.); hlyu@ippcaas.cn (H.Y.)

**Keywords:** gene-edited soybean, rhizosphere microbiome, metagenomics, community structure, functional enrichment

## Abstract

In the present study, we investigated how soybean yield is enhanced upon editing of the gene *GmJAG1* and the consequent influence on the structure and function of the rhizosphere microbiome. Field trials revealed that gene-edited (GE) soybeans had a 55.22% increase in yield without concomitant changes in root length. Metagenomic sequencing of the rhizosphere soil microbiome showed that, compared with the corresponding non-edited line (CK), the alpha diversity of the GE groups remained unaltered, whereas beta diversity differed significantly at the soybean reproductive (R2) stage. Notably, the rhizosphere microbiome of GE soybeans at the R2 stage exhibited enrichment of functional pathways related to transport, amino acid biosynthesis, and central metabolism. These findings suggest that *GmJAG1* editing may shape the functional profile of the rhizosphere microbiome, which could potentially contribute to yield gains. This work offers a novel microbiological perspective for understanding the mechanisms by which yield may be improved in GE crops.

## 1. Introduction

Soybean (*Glycine max*) is one of the most important global food and oil crops and thus holds significant implications for ensuring food security through sustainable production [1]. Although conventional breeding and transgenic technologies have markedly improved soybean yield and stress tolerance, they have also sparked ongoing debates concerning ecological safety [2,3]. In recent years, gene editing technologies, represented by CRISPR/Cas9, have provided a new technical pathway for improving crop genetics owing to their ability to precisely and efficiently target genomic locations and facilitate modifications, typically without introducing foreign genes [4,5,6]. This technology has demonstrated considerable potential for enhancing soybean disease resistance, stress tolerance and yield while optimizing nutritional quality, with the promise of advancing a new generation of sustainable agricultural biotechnology systems [7,8,9].

In terms of genetic improvement of yield traits, gene editing technology offers a novel strategy for achieving increased soybean production through the direct regulation of key agronomic genes, primarily reflected in two aspects. First is the regulation of grain weight and grain number; by editing key genes that control seed size, pods per plant, or seeds per pod, the yield components of soybeans can be directly optimized [10,11,12]. Second is the optimization of plant architecture to enhance yield; through precise modification of genes related to plant height, branching angle and leaf morphology, gene editing enables the development of ideal plant types characterized by compact architecture, robust stems and strong lodging resistance. Such plant types improve field ventilation and light penetration, facilitate high-density cultivation, and consequently significantly increase yield per unit area [13,14,15,16]. For example, editing of the gene *GmJAG1* (encoding a C2H2-type zinc-finger transcription factor) via CRISPR/Cas9 resulted in soybean plants with narrower leaves and increased seed number per pod (particularly 3–4 seeds per pod), ultimately achieving approximately 8% yield improvement in field trials [17,18].

Plant roots are adaptive organs that both influence and are influenced by the physical, chemical and biological properties of the surrounding soil [19,20], including the rhizosphere, which comprises a distinct microbial community (microbiome) that can be influenced by plant roots. The rhizosphere microbiome differs from the general soil microbiome in terms of composition, microbial abundance and functional traits. It both affects and is affected by plant physiology and growth and plays crucial roles in key ecological processes such as nutrient cycling, organic-matter decomposition, disease suppression and plant growth promotion [21,22].

To date, extensive studies have specifically examined the effects of genetically modified crops on rhizosphere microorganisms, with most focusing on transgenic Bt crops or herbicide-tolerant crops [23,24,25]. These studies have generally reported that transgenic events cause negligible or only transient perturbations to rhizosphere microbial community structure. However, whether these conclusions extend to gene-edited crops—especially those with edits in endogenous genes that alter plant architecture and yield components—remains largely unexplored. Unlike transgenic crops that express exogenous insecticidal or herbicide-resistance proteins, GE crops carry precise modifications in endogenous genes, which may lead to more subtle but potentially distinct effects on plant physiology and, consequently, on root–microbe interactions.

How might *GmJAG1*-mediated changes in leaf morphology and seed number per pod influence the rhizosphere microbiome? Although direct experimental evidence is currently lacking, we propose a plausible mechanistic link via change in root exudation. Plant shoot architecture and sink strength (e.g., seed number) are known to influence photosynthetic carbon allocation and partitioning to roots, which in turn affects the quantity and composition of root exudates [22,26]. Changes in root exudates such as organic acids, sugars, amino acids, secondary metabolites can serve as specific signals or substrates that selectively recruit or repel distinct microbial taxa in the rhizosphere [26]. Therefore, we hypothesize that even in the absence of detectable root morphological changes, *GmJAG1*-mediated alterations in plant architecture and yield components may indirectly influence the rhizosphere microbial community by modifying the chemical communication between roots and soil microbes. Testing this hypothesis requires a community-level approach capable of capturing compositional and functional shifts in the rhizosphere microbiome.

Metagenomics is a technique that allows for the direct extraction and high-throughput sequencing of total microbial DNA from environmental samples, followed by bioinformatic analysis, without the need for isolating and culturing all microorganisms present [27,28,29]. This approach is particularly well-suited for examining the compositional and functional changes in rhizosphere microbial communities associated with GE crops. In this context, it is important to distinguish between ecological impacts (i.e., observable changes in microbial community structure and function) and environmental risks (i.e., adverse effects on ecosystem services, biodiversity loss, or unintended ecological harm). Not all microbiome shifts constitute a risk; many may represent neutral or even beneficial plant–microbe associations. A key goal of our study, therefore, is to characterize the nature and magnitude of microbiome changes associated with *GmJAG1*-edited soybeans, thereby providing a foundation for future risk assessment. In this study, *GmJAG1*-edited soybeans were used as experimental material, and metagenomic technology was employed to analyze the correlation between high soybean yield and rhizosphere soil microbial communities. The findings provide a basis for further elucidation of the impact of GE-mediated trait improvement on the rhizosphere microecology and offer theoretical support for the development of microbiome-based strategies to assist in breeding new soybean varieties with high and stable yields.

## 2. Materials and Methods

### 2.1. Plants

The *GmJAG1* GE and CK soybeans lines were obtained from Shandong Shunfeng Biotechnology Co., Ltd. (Jinan, Shandong province, China) and used for all experiments. The CK line is near-isogenic to the edited line except for the targeted 1 bp substitution in the *GmJAG1* locus. Their molecular characteristics were verified by sequencing of PCR products (Figure S1).

The soybeans were planted at the same time in adjacent plots in the experimental field station of the Institute of Plant Protection, Chinese Academy of Agricultural Sciences, Henan Province, China (35°9′31″ N, 113°47′36.46″ E), where wheat had been grown in the previous cropping season. The experiment was conducted in 2025 in a large flat plot where GE soybeans had never been planted. Three replicate plots for each of the two soybean lines were established in a randomized design. Each plot had an area of 20 m^2^ (4 m × 5 m), with a 1 m spacing between adjacent plots. A 0.5 m border row was set up around the outermost plots. Soybeans were planted at a row spacing of 60 cm and a within-row spacing of 8 cm, resulting in a planting density of 208,300 plants per hectare.

### 2.2. Soil Properties and Field Management

The soil in the experimental field is classified as sandy loam, with pH 7.6–8.0, organic matter content ≥ 15 g/kg, available phosphorus ≥ 20 mg/kg, and available potassium ≥ 100 mg/kg. No fertilizer was applied during the soybean growing season and drip irrigation was supplemented as needed based on precipitation. Pesticides were applied at the soybean seedling stage to control *Spodoptera exigua*, and weeds were manually removed. Importantly, the GE and CK soybeans were grown under identical environmental conditions and agronomic management.

### 2.3. Yield Measurement

Yield was measured at the late maturity stage (R8, 23 October). Ten soybean plants were sampled from each plot using the five-point sampling strategy, then manually threshed, air-dried naturally, and weighed.

### 2.4. Rhizosphere Soil Sampling

Rhizosphere soil samples were collected at the third trifoliolate stage (V3, 18 July), full bloom stage (R2, 23 August) and R8 stage (23 October). Fifteen or ten soil samples (V3: 15, R2: 10, R8: 10) were collected from each plot using a five-point sampling strategy and combined into one sample. Thus, a total of 18 samples (2 soybean lines × 3 replications × 3 stages) were collected. To obtain rhizosphere soil, roots with tightly adherent soil were carefully excavated using a trowel. The root system was then vigorously shaken to remove loosely attached soil (considered non-rhizosphere bulk soil). Only soil that remained firmly attached to the root surface after shaking was collected as rhizosphere soil. The roots with this tightly adherent soil were placed in self-sealing bags and transported to the laboratory on ice. The samples were air-dried in a ventilated area [30,31], and soil adhering to the roots was brushed off using a sterilized brush to obtain rhizosphere soil samples. Each sample was then finely sieved through an 80-mesh sieve and stored at −80 °C for future use. After soil collection, root length was measured and recorded.

### 2.5. Profiling of Rhizosphere Soil Microorganisms

#### 2.5.1. Extraction and Shotgun Sequencing of Metagenomic DNA

Samples of total microbial genomic DNA were extracted using the OMEGA Mag-Bind Soil DNA kit (M5635-02, Omega Bio-Tek, Norcross, GA, USA). The quantity and quality of extracted DNA were measured using a Qubit™ 4 Fluorometer (Invitrogen, Carlsbad, CA, USA) and agarose gel electrophoresis, respectively. The extracted microbial DNA was processed to construct metagenome shotgun sequencing libraries with insert sizes of ~400 bp using the TruSeq Nano DNA LT Library Preparation kit (Illumina, San Diego, CA, USA). Each library was sequenced with the Illumina NovaSeq X plus platform with the PE150 strategy by Personal Biotechnology Co., Ltd. (Shanghai, China).

#### 2.5.2. Metagenomics Analysis

Raw sequencing reads were processed to obtain quality-filtered reads for further analysis. The quality control parameters used with fastp were as follows: -l 50 -g -W 5 -5 -q 20 -u 30. No host sequence filtering was performed in this study. First, sequencing adapters were removed from sequencing reads using Cutadapt (v1.2.1). Second, low-quality reads were trimmed using a sliding-window algorithm in fastp (v0.23.2). The resulting quality-filtered metagenomics sequencing reads were classified taxonomically using Kraken2 (v2.0.8-beta) against an integrated database, which included archaeal and bacterial genomes from the GTDB database (release207), viral sequences from the RVDB database (v24.1), and eukaryotic microbial sequences from the NCBI-nt database (1 November 2022). Reads assigned to metazoans or viridiplantae were removed prior to subsequent analysis.

Megahit (v1.1.2) was used to assemble reads in each sample using meta-large with preset parameters. To avoid ambiguous homologous alignments and potential false-positive annotations that are frequently associated with short sequences, contigs shorter than 300 bp were discarded. The remaining contigs (>300 bp) were then pooled and clustered using mmseqs2 with “easy-linclust” mode, setting the sequence identity threshold to 0.95 and covered residues of the shorter contig to 90%. The lowest common ancestor taxonomy of the non-redundant contigs was obtained by aligning them against the NCBI-nt database using mmseqs2 with “taxonomy” mode, and contigs assigned to viridiplantae or metazoa were dropped in the subsequent analysis. Prodigal (V2.6.3) was used to predict genes in the contigs. Coding DNA sequences for all samples were clustered using mmseqs2 with the “easy-cluster” mode, with the protein sequence identity threshold set to 0.95 and covered residues of the shorter contig to 90%.

To assess the relative abundance of genes, the high-quality reads from each sample were mapped onto the predicted gene sequences using Minimap2 and using featureCounts to count the number of reads aligned to gene sequences for each gene. To account for differences in both gene length and sequencing depth, we used Transcripts Per Kilobase per Million mapped reads (TPM) as the normalization method. TPM is calculated as follows:

$$TPM_{i}=\frac{(R_{i}/L_{i})\times 10^{6}}{\sum_{1}^{n}(R_{i}/L_{i})}$$

where Ri represents the raw read count (RC) of gene i in a given sample (i.e., the number of reads mapped to that gene). Li represents the length of gene i in kilobases (kb). The functionality of each non-redundant gene was obtained and annotated using mmseqs2 with the “search” mode against the KEGG protein database and using Diamond (v2.0.15) against the PCyc and NCyc database, respectively. Gene Ontology was assigned using map2slim (www.metacpan.org, accessed on 20 December 2025).

#### 2.5.3. Statistical Analyses

All sequencing data were processed by bioinformatics pipelines on the GENESCLOUD platform (https://www.genescloud.cn, accessed on 22 December 2025). Alpha diversity indices such as the Chao1 richness estimator, Observed species, Simpson index and the Shannon diversity index, were calculated based on the genus-level relative abundance table derived from shotgun metagenomic sequencing using MetaPhlAn 4 (v4.1.0). Statistical differences among groups were assessed using the Kruskal–Wallis test, followed by Dunn’s post hoc test with Benjamini–Hochberg adjustment for multiple comparisons. Beta diversity, assessed using Bray–Curtis distance metrics [32], was visualized via principal coordinate analysis (PCoA) and box plots to investigate compositional and functional variation in microbial communities across samples. PERMANOVA (Adonis) was used to test for significant differences among groups, and pairwise comparisons between GE and CK groups at each growth stage were performed using the Wilcoxon rank-sum test. Based on the taxonomic and functional profiles of non-redundant genes, linear discriminant analysis effect size (LefSe) was performed to detect differentially abundant taxa and functions across groups using default parameters [33], wilcoxon test were performed to assess the differences between GE and CK groups, and differences were considered significant at *p* < 0.05 and linear discriminant analysis (LDA) > 2.5.

### 2.6. Statistical Analysis

Differences in yield and root length between GE and CK soybean plants were analyzed using the Student’s *t* test. Differences were considered significant at *p* < 0.05. Statistical analyses were conducted using the SPSS software package (version 17.0 for Windows, 2008).

## 3. Results

### 3.1. Yield and Root Length

**Figure 1:**
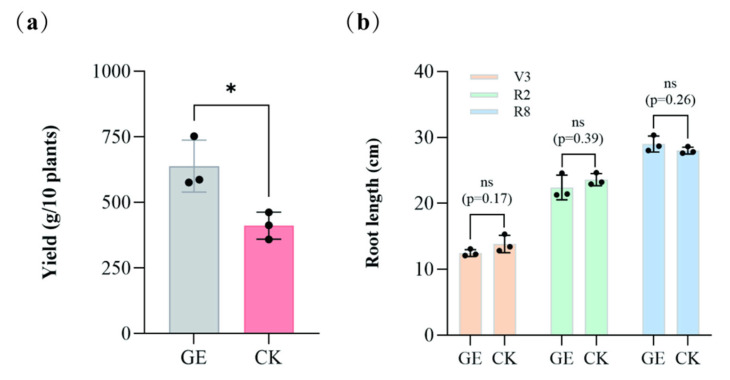
Comparison of yield and root length between gene-edited soybean and its corresponding non-edited line. (**a**) Yield measured at harvest. (**b**) Root length measured at stages V3, R2 and R8. Data represent the mean ± SE (*n* = 3). * Statistically significant difference between the two groups (*p* < 0.05); ns, not significant.

Soybean yield was calculated after the harvest. The yield of GE soybean was significantly higher than that of its corresponding non-edited line (638.73 ± 57.17 vs. 411.50 ± 29.92 g/10 plants, *p* = 0.024; Figure 1a), with an increase of 55.22%. Root length was measured at the V3, R2, and R8 stages of soybean growth; there were no significant differences between the GE and CK soybeans at any of these three stages (Figure 1b).

### 3.2. Microbiota Diversity in Rhizosphere Soil

#### 3.2.1. Microbiota Composition in Rhizosphere Soil

**Figure 2:**
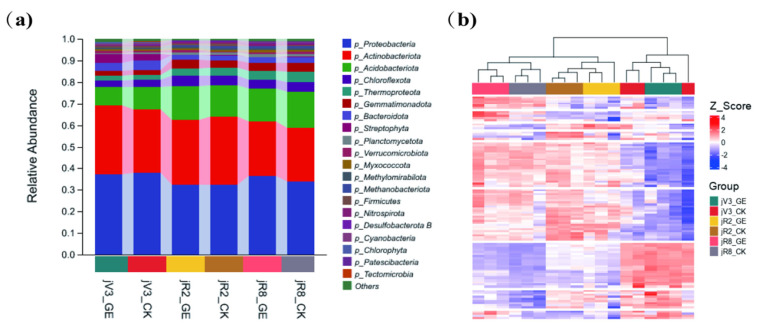
Rhizosphere soil microbial composition across growth stages. (**a**) Bar plot of rhizosphere soil microbial composition at the phylum level. Different colors represent different phyla. (**b**) Heatmap of rhizosphere soil microbial composition at the genus level.

The relative abundance at the phylum level of the microbiota in rhizosphere soil samples taken at different stages of soybean growth, namely V3, R2 and R8, was shown in Figure 2a. Details concerning composition and relative abundance of the rhizosphere soil microbiota at the phylum level during different growth stages is presented in Table 1.

For all growth stages and treatment groups, the top three phyla were consistently *Proteobacteria*, *Actinobacteriota* and *Acidobacteriota*, which together accounted for more than 75% of the total community abundance, forming the most stable core structure of the rhizosphere microbial community (Figure 2a). At the V3 and R2 stages, no significant differences in the relative abundances of the top 20 phyla were observed between the GE and CK groups. At the R2 stage, however, the relative abundances of *Verrucomicrobiota* and *Myxococcota* were significantly higher in the GE group than in the CK group. From V3 to R2, abundance of non-dominant phyla *Streptophyta* decreased sharply from approximately 3–4% to ~0.1%, indicating that its contribution diminished as the crop developed. Concurrently, the abundance of *Acidobacteriota* increased significantly, rising from 8.64 to 10.44% at V3 to approximately 15% at R2 (Table 1).

The distribution of the microbiota community composition at the genus level is illustrated in the heatmap shown in Figure 2b. Replicates of the same treatment generally clustered together, as did samples from the same growth stages.

#### 3.2.2. Alpha Diversity Analysis at Different Growth Stages

**Figure 3:**
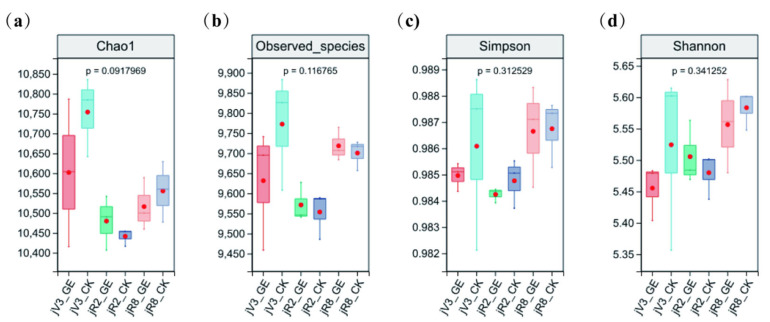
Alpha diversity of the rhizosphere soil microbiota across growth stages. (**a**) Chao1 index, (**b**) Observed species, (**c**) Simpson index, (**d**) Shannon index. Statistical differences among the six groups were assessed using the Kruskal–Wallis test, followed by Dunn’s post hoc test with BH adjustment for multiple comparisons. with *p* values displayed in the upper-middle section of each box. The red dots within boxes denote mean values.

Alpha diversity describes the diversity of species within a particular community or sample. Common indices for its assessment are the Chao1 index (estimating species richness), Observed Species (the actual count of species or operational taxonomic units), the Simpson index (a diversity measure that emphasizes dominant species), and the Shannon index (which integrates both species richness and evenness). The Chao1 and Observed Species indices showed similar trends: stage V3 > R8 > R2. However, no significant differences were observed among the six groups (all *p* > 0.05; Figure 3a,b). The Simpson and Shannon indices also exhibited consistent trends, yet again there were no significant differences among the six groups (all *p* > 0.05; Figure 3c,d).

#### 3.2.3. Beta Diversity Analysis at Different Growth Stages

**Figure 4:**
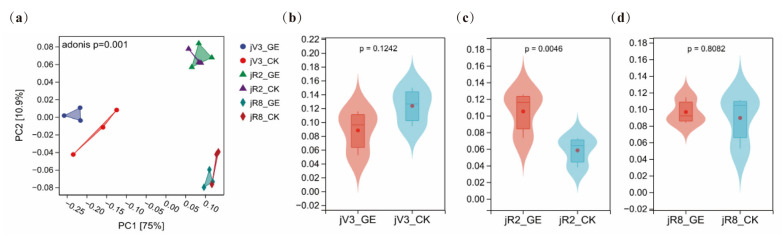
Beta diversity of the rhizosphere soil microbiota at different soybean growth stages. (**a**) Principal coordinate analysis (PCoA) based on Bray–Curtis distances. PERMANOVA (Adonis) revealed significant differences in microbial community composition among the six groups (*p* < 0.01). (**b**–**d**) Pairwise comparisons of Bray–Curtis distances between the gene-edited (GE) and control (CK) groups at three growth stages: (**b**) V3, (**c**) R2, and (**d**) R8. Statistical differences between GE and CK at each stage were assessed using the Wilcoxon rank sum test, with *p*-values indicated above each box plot. Red dots within boxes represent mean values.

Beta diversity is employed to assess the dissimilarity in microbial community structure and composition across various samples. PCoA analysis revealed distinct clustering patterns among the six tested groups. PERMANOVA (Adonis) confirmed significant differences in microbial community composition across the six groups (*p* < 0.01, Figure 4a). We further performed pairwise comparisons between the GE and CK groups at each individual growth stage using the Wilcoxon rank sum test. At the V3 and R8 stage, no significant differences in Bray–Curtis distances were observed between GE and CK (*p* > 0.05, Figure 4b,d). In contrast, at the reproductive stage R2, a significant difference in Bray–Curtis distances was detected between the two groups (*p* < 0.05, Figure 4c).

#### 3.2.4. Differentially Present Microorganisms

**Figure 5:**
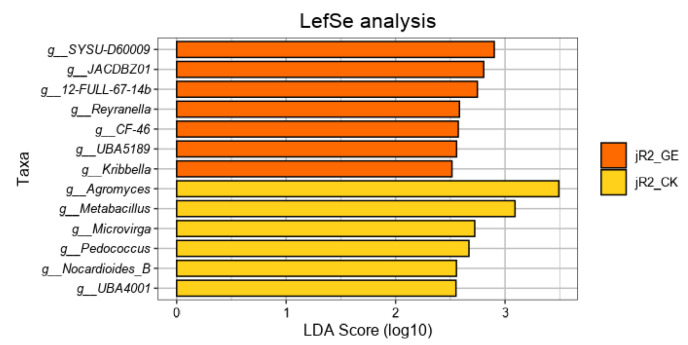
List of discriminant taxa at the genus level at stage R2, identified by LEfSe analysis based on an LDA score threshold of >2.5.

Given the significant differences in beta diversity between the GE and CK groups at stage R2, we subsequently performed an analysis to identify differentially abundant microorganisms between the groups at the R2 stage. LefSe was used to analyze the biomarker genera. At stage R2, the taxonomic composition of rhizosphere microbiota diverged between the two groups. At the genus level, taxa such as *Reyranella* and *Kribbella* were more prominent in GE soybeans, whereas *Agromyces*, *Metabacillus* and others were more abundant in CK soybeans (Figure 5).

#### 3.2.5. Key Functional Enrichment Analysis

**Figure 6:**
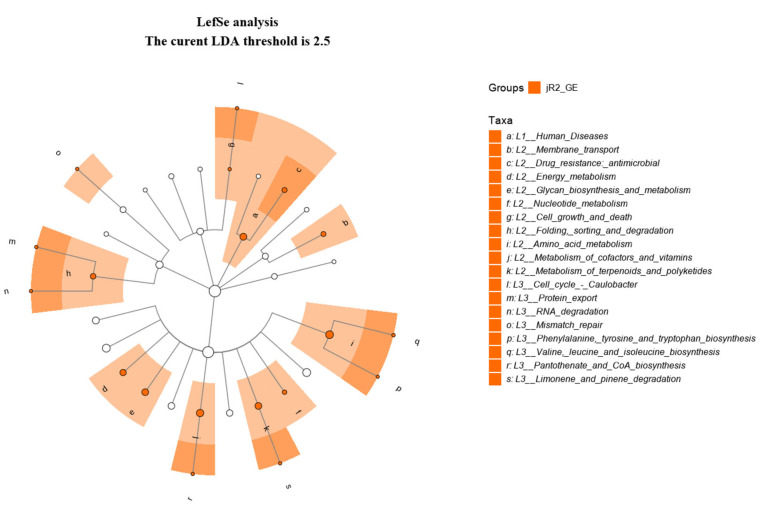
Key functional enrichment analysis of differential microbes through LefSe at stage R2.

To identify functional discriminators between the GE and CK groups at the R2 stage, we conducted LEfSe analysis. This analysis revealed 19 functional features (Figure 6). At KEGG level 2, the enriched categories included membrane transport, drug resistance (antimicrobial), as well as several metabolic pathways such as energy metabolism and amino acid metabolism. At level 3, pathways involved in protein export, RNA degradation, and the biosynthesis of multiple amino acids (e.g., phenylalanine, valine, leucine) were significantly enriched. Collectively, these results suggest that the GE treatment may influence a broad range of microbial functions, particularly those associated with transport, biosynthesis, and central metabolism.

Raw sequencing reads of the rhizosphere soil microbiota were submitted to the NCBI SRA (Submission ID: SUB16205803) and are accessible under BioProject ID PRJNA1469829.

## 4. Discussion

This study systematically investigated the potential association between high-yield traits and changes in the rhizosphere microbiome in GmJAG1-edited soybeans by integrating phenotypic observations with metagenomic analysis. The findings revealed that, against a background of significantly increased yield (+55.22%) without notable changes in root length, the rhizosphere microbial community of GE soybeans exhibited significant differences in beta diversity at the R2 stage. Moreover, these change were associated with a broad range of functional pathways, particularly those related to transport, amino acid biosynthesis, and central metabolism. These results suggest a possible link between trait improvement (via gene editing), root physiology, microbiome composition, and final yield in GE crops.

We used metagenomics to systematically compare the differences in rhizosphere soil microbial community structure between GE and CK soybeans across different growth stages. Compared to traditional methods such as culture-dependent microbial colony counting or 16S/ITS amplicon sequencing, metagenomics has significant advantages in terms of comprehensiveness and depth of information. For example, this approach does not rely on specific primers or probes and directly captures genomic DNA information from all microorganisms in the soil, including bacteria, archaea, fungi, viruses and protists. This largely avoids biases in community structure caused by PCR amplification preferences and is particularly effective for revealing unknown or low-abundance microbial taxa [34,35]. In addition to elucidating microbial taxonomic composition, metagenomics enables direct exploration of their functional potential. By annotating data against functional databases such as KEGG and COG, it allowed for systematic comparison of differential abundance in key metabolic pathways—such as carbon and nitrogen cycling, stress response, and antibiotic resistance—between GE and CK soils, thereby providing critical insights into the underlying mechanisms driving changes in community structure [36].

The gene *GmJAG1*, which is located at the Ln locus, is a major gene regulating seed number per pod and leaf morphology in soybean. A single base-pair substitution from G to C within this locus leads to a change in the amino acid, resulting in loss of GmJAG1 function and the formation of the Ln mutant allele. This mutation significantly alters leaf shape and increases seeds per pod and has been widely used in soybean breeding [17,18,37,38]. Consistent with expectations, the *GmJAG1*-edited soybean in this study exhibited a significant increase in yield. The 55.22% yield increase observed in our study is substantially higher than the approximately 8% increase previously reported for *GmJAG1* editing [17,18]. Many factors could contribute to yield increase, including genetic background, editing sites, environmental conditions, agronomic management, and potential edge effects [39,40,41,42]. In our study, the GE and CK lines were grown under identical environmental conditions and agronomic management. Furthermore, we adopted a five-point sampling method that excluded border rows to minimize edge effects. Therefore, we infer that differences in genetic background and editing sites are the most likely causes of the observed high-yield increase. This is particularly relevant given that editing the same gene can lead to different yield outcomes. For example, Li et al. reported that editing the soybean *GmCKX3* gene resulted in a yield increase of 13–17% [39], whereas Yang et al. achieved a yield increase of over 40% by editing the same gene [40]. Such variability underscores the influence of genetic background and editing strategy on phenotypic outcomes. Notably, our single-year, single-location result may represent an extreme positive response, and further multi-year, multi-location validation is necessary to evaluate the true and stable yield benefit.

Current research findings indicate that there is a close relationship between high crop yield and root length [43], yet longer roots do not necessarily lead to higher yields. Instead, yield potential is jointly determined by root architecture, depth, distribution, physiological activity and the degree of adaptation to the environment [44,45]. In our present study, the substantial increase in yield of GE soybeans was not accompanied by enhanced root physical exploration capacity (root length), suggesting that yield gains likely stemmed primarily from an improvement in the efficiency of root physiological activity or optimization of the rhizosphere microenvironment rather than mere morphological expansion. However, we note that root phenotyping was restricted to length measurement, comprehensive architectural traits such as root biomass, surface area, branching, or diameter were not assessed. Future controlled experiments with full root system characterization are needed to determine whether *GmJAG1* editing affects additional root parameters beyond length.

This study revealed that the effects of gene editing on the microbiome exhibited growth-stage specificity and functional selectivity. At the phylum level, stage R2 was characterized by the enrichment of *Myxococcota* (obligate predators, potentially regulating community structure) and *Verrucomicrobiota* (capable of polysaccharide degradation and mucosal metabolism) [46,47]. This temporal community succession suggests that GE soybeans may undergo stage-specific domestication of rhizosphere microbes, likely mediated by growth-stage-dependent alterations in the composition and quantity of root exudates [26,48]. At the genus level, the abundance of *Kribbella,* a member of phylum *Actinomycetota,* was significantly higher in GE soybeans than in CK soybeans at R2 stages. Previous research indicates that *Kribbella* can colonize plant roots and may promote plant growth by suppressing pathogen development and facilitating nutrient uptake [49,50]. Results of this study demonstrate that plants can enrich specific bacterial groups with plant growth-promoting potential from the soil microbial pool into their root systems, a finding consistent with research conducted on other plants [51,52]. Furthermore, some bacterial taxa with unknown function were identified and require further study.

Metagenomic functional analysis provided predictive insights into microbial potential. During stage R2 (a critical period for yield formation), the rhizosphere microbial community of GE soybeans exhibited higher predicted relative abundances of a broad range of microbial functions, particularly those related to transport, amino acid biosynthesis, and central metabolism. The enrichment of membrane transport pathways may indicate enhanced nutrient uptake capacity of the rhizosphere microbiome under GE treatment, while the enrichment of antimicrobial resistance-related genes may reflect microbial adaptation to rhizosphere stresses [53,54]. Notably, the enrichment of amino acid biosynthesis pathways (e.g., phenylalanine, valine, leucine) may be linked to the yield improvement of GE soybeans, as these amino acids serve as precursors for protein synthesis and plant secondary metabolism [55]. Furthermore, the enrichment of RNA degradation and protein export pathways suggests an enhanced protein secretion capacity of the GE-associated microbiome, which may help modulate the rhizosphere microenvironment [56]. Based on these correlative findings, we hypothesize that GE soybean roots may enrich a microbial community with distinct predicted functional attributes. However, whether this translates into actual differences in ecological service efficiency within the root space, or directly contributes to the observed yield increase, remains to be tested through causal experimental approaches.

In contrast to traditional transgenic crops (which typically introduce exogenous genes for insect resistance or herbicide tolerance), the GE approach in this study (precise knockout of endogenous genes to optimize plant architecture and seed set) had a significant yet qualitatively distinct impact on the microbiome. Previous studies have often reported that transgenic crops have negligible or transient effects on microbial diversity [57,58,59,60]; in our study, however, the GE soybeans did not alter alpha diversity but significantly altered community structure (beta diversity) and strongly enriched specific functions. This suggests that the ecological effects of our GE soybeans may focus more on functional remodeling rather than species survival or loss. From an ecological safety perspective, if such a functionally specialized microbial community could be stably maintained, it may constitute a novel micro-ecological balance, with its enhanced nutrient cycling and stress resistance functions potentially yielding positive environmental effects. However, its long-term stability, whether it leads to reduced functional redundancy, and potential changes in susceptibility to soil-borne diseases require further long-term monitoring and observation.

This study has several limitations. First, the number of biological replicates was modest, with only three plots per treatment, and yield data were obtained from only 10 soybean plants per plot. Second, comprehensive root architectural traits, such as biomass, surface area, branching, or diameter were not assessed. Third, our functional inferences are based solely on predicted abundance from metagenomic annotation, which reflects the potential of the microbiome rather than actual in situ activity. Furthermore, we did not measure root exudate chemistry, leaving it unknown which specific molecules (e.g., simple sugars, organic acids, flavonoids, or signaling compounds) are responsible for recruiting enriched microbial taxa. To address these limitations and further validate our findings, future studies could consider the following aspects: (1) conducting multi-location and multi-year replicated cropping trials to evaluate the stability of the observed yield improvement and microbiome patterns, and to test whether enhanced functional specialization reduces functional redundancy; (2) isolating key functional strains and directly verifying their growth-promoting effects; (3) collecting root exudates at the R2 stage and integrating metabolomics to directly identify key exudate molecules that drive specific microbial recruitment [61]; and (4) integrating multi-omics data (e.g., transcriptomics, metabolomics) to construct a systematic regulatory network linking gene editing, plant physiology, and microbial function.

## 5. Conclusions

*GmJAG1*-edited soybeans exhibited a 55.22% yield increase over CK soybeans. At the R2 stage, the GE line was associated with significant beta-diversity differences and enrichment of transport, amino acid biosynthesis, and central metabolism pathways in the rhizosphere microbiome. These findings suggest that *GmJAG1* editing may shape the functional profile of the rhizosphere microbiome, which could potentially contribute to yield gains. However, as these results are based on predicted functional abundances, experimental validation is needed to confirm these predicted functional changes.

## Figures and Tables

**Table 1:** The composition and relative abundance of rhizosphere soil microbiota at the phylum level during different growth stages. (%, *n* = 3).

Phylum	V3		R2		R8	
	GE	CK	GE	CK	GE	CK
*p_Proteobacteria*	37.02 ± 0.99	37.88 ± 2.25	32.35 ± 1.78	32.28 ± 0.92	36.32 ± 2.42	33.69 ± 1.35
*p_Actinobacteriota*	32.17 ± 1.33	29.23 ± 1.28	30.24 ± 2.06	31.55 ± 0.61	25.42 ± 0.70	25.12 ± 0.25
*p_Acidobacteriota*	8.64 ± 0.96	10.44 ± 1.50	15.30 ± 1.62	14.79 ± 1.04	15.17 ± 0.84	16.79 ± 0.87
*p_Bacteroidota*	3.99 ± 0.19	4.23 ± 0.66	2.33 ± 0.18	2.18 ± 0.11	2.61 ± 0.28	2.90 ± 0.09
*p_Streptophyta*	3.80 ± 0.53	2.96 ± 0.68	0.10 ± 0.05	0.15 ± 0.01	0.03 ± 0.01	0.03 ± 0.01
*p_Chloroflexota*	3.00 ± 0.14	3.45 ± 0.62	5.09 ± 0.50	4.46 ± 0.23	4.00 ± 0.22	4.33 ± 0.05
*p_Thermoproteota*	2.17 ± 0.25	2.31 ± 0.45	3.32 ± 0.47	3.70 ± 0.04	4.38 ± 0.30	4.66 ± 0.45
*p_Gemmatimonadota*	1.99 ± 0.40	2.28 ± 0.34	3.90 ± 0.60	3.31 ± 0.21	3.59 ± 0.47	4.13 ± 0.32
*p_Verrucomicrobiota*	1.08 ± 0.20	0.95 ± 0.08	0.83 ± 0.03 *	0.67 ± 0.06	0.85 ± 0.03	0.85 ± 0.08
*p_Myxococcota*	0.72 ± 0.05	0.68 ± 0.07	0.97 ± 0.10 *	0.72 ± 0.06	1.05 ± 0.24	0.81 ± 0.04
*p_Planctomycetota*	0.71 ± 0.06	0.80 ± 0.13	1.17 ± 0.09	1.08 ± 0.03	1.30 ± 0.04	1.38 ± 0.03
*p_Cyanobacteria*	0.67 ± 0.28	0.86 ± 0.45	0.12 ± 0.01	0.13 ± 0.01	0.11 ± 0.00	0.12 ± 0.01
*p_Chlorophyta*	0.65 ± 0.09	0.54 ± 0.12	0.02 ± 0.01	0.02 ± 0.00	0.01 ± 0.00	0.01 ± 0.00
*p_Firmicutes*	0.63 ± 0.06	0.56 ± 0.08	0.68 ± 0.35	1.15 ± 0.17	0.33 ± 0.01	0.33 ± 0.01
*p_Methylomirabilota*	0.53 ± 0.05	0.65 ± 0.12	0.66 ± 0.02	0.70 ± 0.07	0.92 ± 0.08	0.91 ± 0.05
*p_Desulfobacterota B*	0.30 ± 0.02	0.31 ± 0.06	0.44 ± 0.05	0.50 ± 0.01	0.55 ± 0.01	0.56 ± 0.08
*p_Nitrospirota*	0.27 ± 0.01	0.30 ± 0.04	0.59 ± 0.02	0.63 ± 0.02	0.87 ± 0.04	0.88 ± 0.05
*p_Methanobacteriota*	0.17 ± 0.02	0.18 ± 0.03	0.89 ± 0.16	1.01 ± 0.27	0.86 ± 0.15	0.93 ± 0.33
*p_Tectomicrobia*	0.07 ± 0.01	0.07 ± 0.02	0.11 ± 0.01	0.12 ± 0.00	0.14 ± 0.01	0.12 ± 0.01
*p_Patescibacteria*	0.05 ± 0.00	0.07 ± 0.01	0.11 ± 0.02	0.11 ± 0.02	0.40 ± 0.12	0.41 ± 0.15
*Others*	1.37 ± 0.10	1.25 ± 0.06	0.79 ± 0.03	0.74 ± 0.03	1.10 ± 0.07	1.07 ± 0.01

Note: Student’s *t*-test was used for comparisons at each time point. The asterisk (*) indicates a significant difference (*p* < 0.05) between treatments within a row at the given time point.

## Data Availability

All data generated or analyzed during this study can be found in the article, further inquiries can be directed to the corresponding author.

## Supplementary Materials

The following supporting information can be downloaded at: https://www.mdpi.com/article/10.3390/plants15121828/s1, Figure S1: The mutation of *GmJAG1* was verified by Sanger sequencing.
