# Supplementary material for: Enhanced Yield of GmJAG1-Edited Soybeans Accompanied by Improved Function of the Rhizosphere Microbiome
Source: Plants (Basel). 2026 Jun 12;15(12):1828. doi: 10.3390/plants15121828 (PMC13306578; doi:10.3390/plants15121828)
Supplement: Supplementary file 1 [file plants-15-01828-s001.zip › plants-4337016-supplementary.pdf]

Figure S1

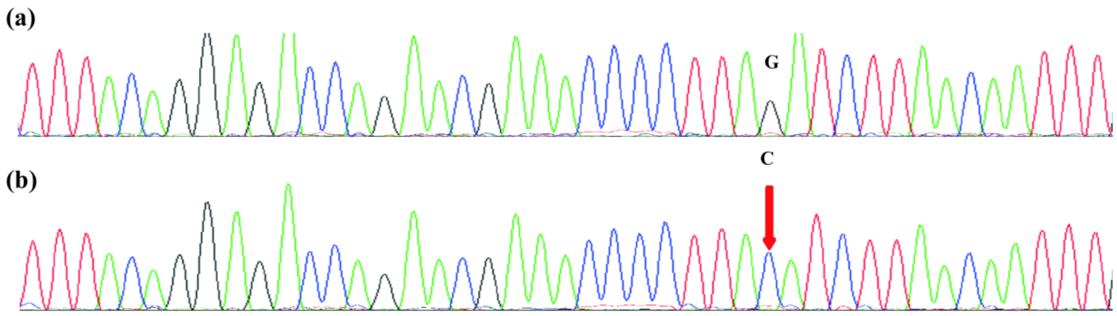

Figure S1. The mutation of *GmJAG1* was verified by Sanger sequencing. **(a)** CK; **(b)** GE. Red arrows indicate mutation locations.
